# The role of Th17 cells in endocrine organs: Involvement of the gut, adipose tissue, liver and bone

**DOI:** 10.3389/fimmu.2022.1104943

**Published:** 2023-01-16

**Authors:** Changyan Zi, Die Wang, Yongxiang Gao, Lisha He

**Affiliations:** ^1^ School of Clinical Medicine, Chengdu University of Traditional Chinese Medicine, Chengdu, China; ^2^ School of International Education, Chengdu University of Traditional Chinese Medicine, Chengdu, China; ^3^ School of Basic Medical Sciences, Chengdu University of Traditional Chinese Medicine, Chengdu, China

**Keywords:** Th17 cells, gut, adipose tissue, liver, bone, endocrine organs.

## Abstract

T Helper 17 (Th17) cells are adaptive immune cells that play myriad roles in the body. Immune–endocrine interactions are vital in endocrine organs during pathological states. Th17 cells are known to take part in multiple autoimmune diseases over the years. Current evidence has moved from minimal to substantial that Th17 cells are closely related to endocrine organs. Diverse tissue Th17 cells have been discovered within endocrine organs, including gut, adipose tissue, liver and bone, and these cells are modulated by various secretions from endocrine organs. Th17 cells in these endocrine organs are key players in the process of an array of metabolic disorders and inflammatory conditions, including obesity, insulin resistance, nonalcoholic fatty liver disease (NAFLD), primary sclerosing cholangitis (PSC), osteoporosis and inflammatory bowel disease (IBD). We reviewed the pathogenetic or protective functions played by Th17 cells in various endocrine tissues and identified potential regulators for plasticity of it. Furthermore, we discussed the roles of Th17 cells in crosstalk of gut-organs axis.

## 1 Introduction

T lymphocytes can be divided into CD4^+^ T cells and CD8+T cells. CD4^+^ T cells are central to the functioning of the entire immune system. CD4^+^ T cells polarize to different phenotypes in a variety of tissues, including T Helper 17 (Th17) cells, Foxp3+T regulatory cells (Treg), Th1 cells, Th2 cells, T follicular helper (Tfh), Th9 and Th22 cells (1). Th17 cells have unique properties and versatile functions. On the one hand, Th17 cells play vital roles in the etiopathogenesis of various inflammatory diseases. On the other, the adaptive cellular immune responses are reinforced by Th17 cells against extracellular bacteria, fungi, and viruses (2). Increased attention has been attracted by Th17 cells recently in the regulation of chronic inflammation and they further participate in metabolic disorders. The plasticity of Th17 cells is influenced by multiple factors, such as cytokine milieu, microbial products and products of metabolism, and Th17 cells exert pathogenicity *via* various mechanisms in endocrine organs.

In addition to classic endocrine organs or tissues, plenty of novel endocrine organs were observed in the past few decades. Gut is regarded as a full-blown endocrine organ, as the gut microbiota make a far-reaching influence on the intestinal milieu (3). Gut plays a pivotal role in the endocrine system by interacting with other endocrine organs, including adipose tissue, liver and bone. Adipose tissue, as endocrine organ, is characterized by complicated and dynamic. Adipose tissue consist mainly of adipocytes as well as connective tissue matrix, nerve tissue, stromovascular cells, and immune cells (4). These components work together as a whole unit (4). Indeed, the endocrine system is marked by the production of hormones, and some parts of liver pathology are in accordance with this property (5). The liver, as a key regulator of metabolic homeostasis, perceives and integrates hormone signals and whole body energy status that are triggered by alterations in metabolism (6, 7). In the past, the skeleton that was considered as the regulator of calcium-phosphorus and haematopoiesis homoeostasis, has now been identified as a crucial modulator of metabolism (8). A large number of groundbreaking research confirm that the bone is a real endocrine organ (8).

**Figure 1:**
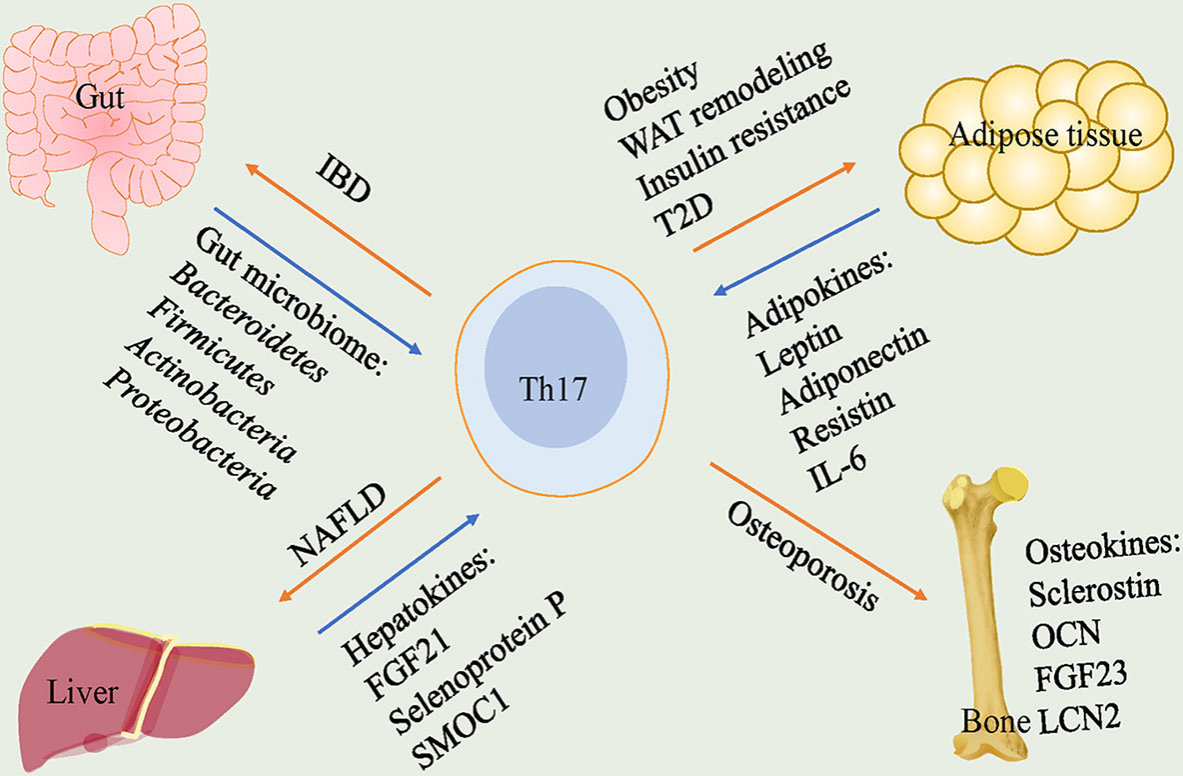
Th17 cells play pathogenetic functions in gut, adipose tissue, liver and bone. In gut, gut microbiome including Bacteroidetes, Firmicutes, Actinobacteria and Proteobacteria lead to a high frequency of Th17 cells that cause IBD. In adipose tissue, adipokines including leptin, adiponectin, resistin and IL-6 participate in the regulation of Th17 cells. Th17 cells trigger obesity, WAT remodeling, insulin resistance and T2D. In liver, hepatokines, FGF21, selenoprotein P and SMOC1, are correlated with levels of Th17 cells and Th17 cells play a crucial role in NAFLD. In bone, Th17 cells are responsible for promoting osteoporosis. Th17, T helper 17; IBD, inflammatory bowel disease; WAT, white adipose tissue; T2D, type 2 diabetes; FGF21, fibroblast growth factor 21; SMOC1, sparc-related modular calcium-binding protein 1; NAFLD, nonalcoholic fatty liver disease; OCN, osteocalcin; FGF23, fibroblast growth factor 23; LCN2, lipocalin-2.

**Figure 2:**
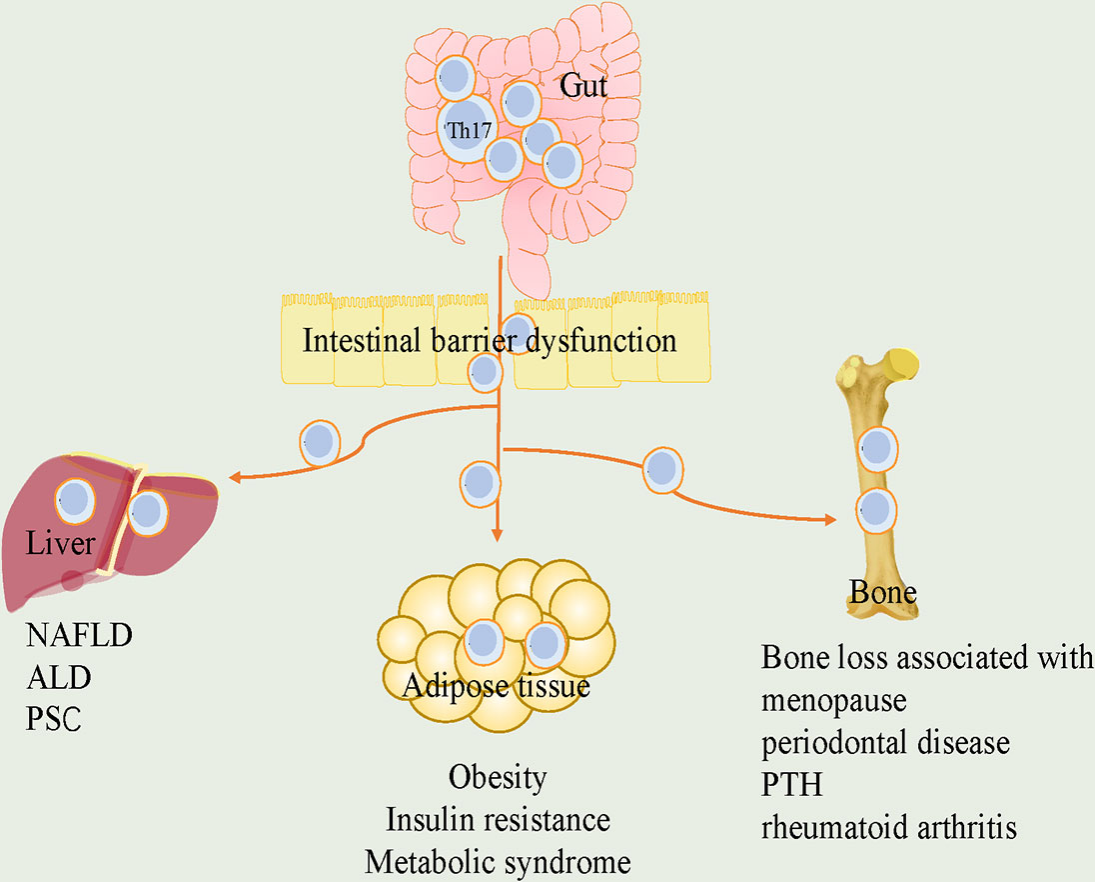
The roles of Th17 cells in the crosstalk of gut and adipose tissue, liver and bone. Loss of gut integrity increases influx of Th17 cells into metabolic tissue. In gut-adipose, due to increased gut permeability, Th17 cells are disseminated from intestine into the adipose. it further stimulates local Th17 cells and contributes to the development of obesity, insulin resistance and metabolic syndrome. In gut-liver, intestinal barrier defects promote liver disease. Th17 cells in intestinal dysbiosis are associated with liver diseases, including NAFLD, ALD and PSC. In gut-bone, intestinal Th17 cells flow into bone, which are critical for bone loss associated with menopause, periodontal disease, PTH and rheumatoid arthritis. Th17, T helper 17; NAFLD, nonalcoholic fatty liver disease; ALD, alcohol-associated liver disease; PSC, primary sclerosing cholangitis; PTH, parathyroid hormone.

As such, the plasticity and adaptability of Th17 cells in response to various stimuli make them attractive targets. We reviewed the pathogenetic or protective functions played by Th17 cells in various endocrine tissues and identified potential regulators for plasticity of it (**Figure 1**). Furthermore, we discussed the roles of Th17 cells in crosstalk of gut-organs axis (**Figure 2**).

## 2 Endocrine organs

The endocrine system is a complex interconnected system of organs. The primary endocrine organs contain hypothalamus, pituitary, thyroid, pancreas, and ovaries or testes, whose primary function is hormonal production and secretion. Over the past few decades, it is well established that secondary endocrine organs (e.g., gut, adipose tissue, liver and bone) also contribute to the regulation of metabolic homeostasis and inflammatory conditions through secretion of a great deal of hormones, proteins and cytokines.

### 2.1 Gut

The gut microbiome contains a large amount of information. Secretin, gastrin and cholecystokinin were identified as gut hormones for the first time (9). Nowadays, with the discovery of multiple gut hormone genes and bioactive peptides, gut is recognized as the largest endocrine organ in the body (9). It is well known that humans have trillions of microorganisms. A union made up of bacteria, fungi, protozoa, and viruses live in several areas of the body, including the lungs, urinary tract vagina, and skin, however, the largest microbial flora exists in the intestine (10). Increasing evidence has suggested that these microbes almost play the role of an extra organ by actively participating in shaping and sustaining our physiological functions (3, 11). Nowadays, it is generally believed that the metabolic ability of the gut microbiota is far superior to that of the human host (12). Interactive diversity between the gut microbiota and the pathophysiology of the host makes it clear that the gut microbiota not only affects the intestinal milieu but also has ability to influence distant tissues and pathways (13).

### 2.2 Adipose Tissue

The discovery of leptin established the status of adipose tissue as an indispensable endocrine organ in 1994 (14). Since then, researches have discovered numerous peptide and non-peptide hormones derived from adipose tissue with autocrine, paracrine or endocrine (15), which make the adipose tissue a complex and dynamic endocrine organ. Adipose tissue not just reacts to afferent signals from classical hormone systems and the central nervous system. It also secretes factors that exert essential endocrine functions (16). Adipose tissue displays a wide range of biological functions, including the regulation of energy consumption, appetite control, glucose homeostasis, insulin sensitivity, inflammation and tissue repair by releasing endocrine factors (17). In addition to the traditional polypeptide adipokines and cytokines, extracellular vesicles (EVs) that are released from a variety of cell types in adipose tissue also mediate the crosstalk between cells and organs (18).

### 2.3 Liver

The liver is a highly active organ that plays key roles in numerous pathological processes, the secretions from liver are well known to have an influence on glucose and lipid metabolism. The role of liver as a crucial secretory organ has long been appreciated (19), which has multiple endocrine functions, including production of hormone and hepatokine, hormone metabolism, synthesis of binding proteins (20). Proteins that are secreted from hepatocytes can affect the process of metabolism through autocrine, paracrine and endocrine signals (21). These proteins include sex hormone-binding globulin (SHBG), fibroblast growth factor 21 (FGF21) and adropin (21). Similar to the functional proteins that are derived from adipose tissue and skeletal muscle, these liver-derived proteins are called hepatokines (22). On account of its anatomy, the liver might play a crucial role in interorgan crosstalk. The liver participates in reapportionment of hormones, lipoproteins, carbohydrates as well as fatty acids to other tissues, as it receives a dual blood supply from the hepatic portal vein and the hepatic arteries (23). Furthermore, the location of “open-pore” sinusoids is between hepatocyte planes, which let exchange of hepatokines (23).

### 2.4 Bone

Bone has classically been known as a “dull” organ over the years. Most recent, from evidence that the skeleton behaves as an endocrine organ capable of influencing bone metabolism and physical stability through the secretion of cytokines (24, 25). Studies have revealed the novel endocrine functions of bone, and bone secretes cytokines that can regulate glucose, energy and phosphate metabolism (26). It has been discovered that osteocytes have a variety of functions, such as remodeling the bone by modulating both osteoclast and osteoblast activity, and also acting as an endocrine cell, which releases factors to sustain skeletal homeostasis through endocrine and paracrine functions (27). Bone-derived cytokines or hormones, such as sclerostin, osteocalcin (OCN), fibroblast growth factor 23 (FGF23) and lipocalin-2 (LCN2), target cells on the bone surface and distant tissues, such as kidney, muscle, and other tissues (28, 29).

## 3 Th17 in endocrine organs

### 3.1 Th17 and Gut

There are two different subsets of Th17 cells in the intestine, and their metabolic mechanisms and inflammatory outputs are different. Homeostatic Th17 cells have a mild inflammatory property, which is a defender of intestinal barrier, while pathogenic Th17 cells secrete plenty of inflammatory cytokines that trigger severe inflammatory response (30). Th17 cells exist in the whole intestinal lamina propria and maintain intestinal homeostasis in a microbiome-dependent manner. By stimulating antigen-activated T cells with TGF-β and IL-6 or IL-21, which can induce STAT3 phosphorylation, the homeostatic Th17 cells can be differentiated (31). Countless factors including microbiome, proteins, and transcription factors influence Th17 cells development and plasticity in the gut and render it pathogenic (32).

Gut is a complicated ecosystem that is composed of intestinal epithelium, immune cells, mucus layer, and resident microbiota. The gut microbiome is the microbial community colonizing the gut, including dominant bacteria, archaea, protists, fungi, and viruses (33). In the adult gut, over 90% of the bacteria are the phyla of *Firmicutes* and *Bacteroidetes* (34). Increasing evidences have shown that T cell subsets in the intestine dynamically present the signals of microbiota (35). Particular components of the gut microbiome take part in the production of Th17 cells and subsequent production of inflammatory response (33).

Intestinal draining lymph nodes are located in the mesentery of the small intestine and colon, where adaptive immune responses are further formed by the intestinal symbionts, and naïve T cells are differentiated within the mesenteric lymph nodes (MLNs) (36). As for differentiation of CD4^+^ T cells, especially Th17 cells that have a preference for the gut, firstly, dendritic cells reach MLNs, then they interact with naïve T cells and stimulate them (36). Dysbiosis can result in damage of immune responses with destruction of mucosal barriers (37). Migration of gut microbiome to the MLNs and change of the cytokine milieu in intestinal mucosa make MLNs tend to an inflammatory phenotype with activation of Th17 cells, which lead to the inflow of neutrophils and give rise to a severe inflammatory state (37).

Inflammatory bowel disease (IBD), including Crohn’s disease and ulcerative colitis, is a chronic inflammatory disease. A number of metabolic disorders have been proposed to be related to IBD. Studies have revealed analogies of pathophysiological characteristics between metabolic disorders and IBD, including adipose tissue disorder, immune response dysfunction, chronic inflammation and liver metabolic disorder (38, 39).

Researches have showed that IBD is closely connected with the immune response and may alter intestine barrier function that is resulted from gut microbiome disorder (40). Although the mechanism of bacterial reactive pathogenic T cells in IBD is still not completely clear, bacteria-reactive CD4^+^ T cells gather in the intestinal mucosa of patients with IBD and are considered to play a key role in the pathogenesis of diseases (41). Among these CD4^+^ T cells, Th17 cells have been found to accumulate in the intestinal mucosa and are reactive to resident bacteria (42). The level of Th17 cells induced by gut microbiota was a prediction of IBD and stated the severity of disease in the Rag1-/- colitis model (43). Bacteria including those that adhere to epithelial cells and several clostridium species can alter differentiation of Th17 cells and segmented filamentous bacteria (SFB) are the main driving force of Th17 responses (44, 45).

In terms of bacterial composition at the phylum level, *Bacteroidetes, Firmicutes, Actinobacteria* and *Proteobacteria* are closely related to pro-inflammatory Th17 cells in IBD. Studies have exhibited a relatively low abundance of *Firmicutes* phylum (46, 47) and *Bacteroidetes* (46, 48, 49), which showed a high frequency of Th17 cells in IBD. However, a relatively increased abundance of the *Firmicutes* phylum (48) and *Bacteroidetes* (47) was observed in IBD, which also enhanced Th17 cells. Luo et al. (47) and Tong et al. (49) reported that *Proteobacteria* displayed a relatively high abundance in ulcerative colitis with increase of Th17 cells. In addition, totally opposite roles of *Actinobacteria* (47, 48) that increased the level of Th17 cells, was found in ulcerative colitis.

At genus level, a relatively high abundance of *Bacteroides* (40, 46), *Actinobacterium Eggerthella lenta* (50) and *Bacteroides ovatus* (51) was found in IBD, which induced high levels of intestinal pro-inflammatory Th17 cells. *Lachnospiraceae* (40), *Alistipes* (40), *Lactobacillus* (40), *Bilophila* (40), *Desulfovibrio* (40), *Alloprevotella* (46), *Butyricicoccus pullicaecorum* (47), *Enterorhabdus* (49) and *unclassified_Bacteroidia* (48) displayed a relatively low abundance, which resulted in a higher frequency of pathogenic Th17 cells in a gut microbiota-dependent manner in IBD. In addition, a relatively high abundance of *Bifidobacterium* (48) but a relatively low abundance of *Lactobacillus* (52), *Oscillibacter* (52), *Pseudoflavonifractor* (52), *Clostridium XIVa* (52), *Johnsonella* (52) and *Rothia* (52) were found to promote Th17 cells on different diet. Treatment of microbiota-reactive CD4^+^ T cells with IL-1β, IL-6, or IL-23 during stimulation with *E coli*, *S typhimurium*, *L acidophilus*, or *B animalis* caused an increase in IL-17A production, which suggested that these cytokines might promote Th17 polarization in bacteria-reactive manner (53). Research findings on the gut microbiota and pathogenic Th17 cells are summarized in **Table 1**.

**Table 1:** .

Author	Year	Disease	Gut microbiome		Contribution to pathogenic Th17 cells
			Phylum	Genus	
Liu (46)	2020	Colitis	Bacteroidetes↓ Firmicutes↓	Alloprevotella↓ Bacteroides↑	↑
Ang (48)	2020	/	Bacteroidetes↓ Actinobacteria↑ Firmicutes↑	Bifidobacterium↑	↑
Luo (47)	2019	Ulcerative Colitis	Firmicutes↓ Actinobacteria↓ Proteobacteria↑ Bacteroidetes↑	Butyricicoccus pullicaecorum↓	↑
Tong (49)	2021	Ulcerative Colitis	Bacteroidetes↓ Proteobacteria↑	Enterorhabdus↑ unclassified_Bacteroidia↑	↑
Zhang (40)	2019	Colitis	/	Bacteroides↑ Lachnospiraceae↓ Alistipes↓ Lactobacillus↓ Bilophila↓ Desulfovibrio↓	↑
Alexander (50)	2022	Colitis	/	Actinobacterium Eggerthella lenta↑	↑
Wilck (52)	2017	/	/	Lactobacillus↓ Oscillibacter↓ Pseudoflavonifractor↓ Clostridium XIVa↓ Johnsonella↓ Rothia↓	↑
Lavoie (51)	2019	Crohn's disease	/	Bacteroides ovatus↑	↑
Hegazy (53)	2017	IBD	/	E coli S typhimurium L acidophilus B animalis	↑

Moreover, the hormones secreted from intestinal epithelium also have significant impact on Th17 function. The colonic epithelium plays a role in innate and adaptive mucosal immunity and form a mucus barrier (54). Serum amyloid A (SAA) proteins that promote adaptive immunity, are family of retinol-binding proteins expressed in the intestinal epithelium and liver (55, 56). In the differentiation of Th17 cells, IL-6 can combine with the SAAs instead of TGF-β, which result in a pathogenic pro-inflammatory Th17 cell differentiation program (57). In addition to promote local Th17 cell proliferation and/or retention, SAA may promote Th17 cytokine production, such as IL-17 (58).

Glucagon-like peptide-1 (GLP-1) is an incretin hormone secreted from enteroendocrine cells that reside within intestine epithelium (59). Targeting GLP-1 may be an effective therapeutic strategy for Th17/Treg-mediated inflammatory diseases (60). A clinical research showed that GLP-1 appeared to decrease production of Th17-related cytokines in people with obesity and asthma (61).

### 3.2 Th17 and Adipose Tissue

Adipose tissue (AT), as endocrine organ, is a crucial regulator of excessive fat storage, energy intake and consumption through releasing multiple factors, including adipokines, chemokines and cytokines (62). From the cell perspective, AT contains only 10-30% adipocytes, while the rest of the tissue is made up of various cells, which is called the stromal vascular fraction (SVF) (63), a producer of a series of metabolic diseases. Adipose tissue divides into three types, including white adipose tissue (WAT), beige adipose tissue and brown adipose tissue (BAT). White adipose tissue which mainly lies in subcutaneous or intravisceral sites, is the major energy store. Brown adipose tissue, that locates in the interscapular region, is responsible for energy consumption. Beige adipose tissue are scattered in WAT and can present a brown-like phenotype (64). These three types of adipose tissues also have endocrine functions and play major roles in whole body metabolism especially in obesity.

In 1993, the concept of AT inflammation was first proposed in *Science* (65). AT inflammation is largely caused or exacerbated by the following factors, including immune cell recruitment rapidly, remodeling of the AT stromal immune components (e.g., immune cells, endothelial cells, fibroblasts and adipocyte progenitors), and AT immune cell dysfunction (66). Plentiful experiments have demonstrated that adipose tissue expansion brings about a complicated and extensive immune response, including innate and adaptive immune system, which play essential roles in the modulation of inflammation (67). The infiltration of immune cells into the AT, including macrophages, dendritic cells, neutrophils, T and B cells, give rise to AT inflammation in obesity (68). Studies have shown that the T cells that are skewed towards a Th17 phenotype, generally accumulate in obese adipose tissue in humans and mice (69–72). Moreover, most recent finding has suggested that obesity convert the classical Th2-predominant disease to a more severe disease with prominent Th17 inflammation (73).

Adipocytes are related to production of Th17 cells, including antigen presentation, costimulatory molecule and further proliferation and differentiation. Major histocompatibilty complex (MHC) class II molecules and costimulators, which are regarded as antigen-presenting cells (APCs) to promote the activation of CD4^+^ T cells, are overexpressed in obese adipocytes (74). In mice, adipocyte MHCII increased within 2 weeks on high-fat diet (HFD) and adipocytes can activate T cells directly *via* the MHCII pathway (75). Large adipocytes, which highly expressed MHCII and stimulated CD4^+^ T cells as APC, were the major contributors to adipose tissue inflammation (76). CD40 (74), CD80 (74), CD86 (74) and OX40 (77) were induced in adipocytes of obese human or mice, and may co-stimulate adipose resident T cells in obesity. Additionally, adipose tissue, as a key endocrine organ, is a modulator of Th17 cells differentiation and pathogenicity by secreting plentiful bioactive substances, termed adipose-derived secreted factors or adipokines.

The family of adipokines is very broad, including leptin, adiponectin, resistin, IL-6 and hundreds more (78, 79). What’s more, some novel adipokines were proposed in recent two years, including Isthmin 1 (80) and leucine-rich alpha-2-glycoprotein 1 (LRG1) (81), which played an active role in the regulation of hepatosteatosis, insulin resistance, glucose uptake and glucose tolerance. However, interaction between these novel adipokines and Th17 cells requires further exploration.

#### 3.2.1 Leptin

Leptin is a pro-inflammatory factor that is involved in both adaptive and innate immunity (82). Increasing evidences have suggested that leptin promotes Th17 cells differentiation (83–85) and this process may be achieved by NF-κB, MAPK, JNK pathway and β3 integrin receptor (86). Furthermore, leptin polarized Th17 cells indirectly through the secretion of pro-inflammatory cytokines, such as IL-6, IL-12, TNF-α (85) and ILC2, which promoted retinoic acid-related orphan receptor γt (RORγt) expression (a transcription factor for Th17 cell differentiation) (87).

#### 3.2.2 Adiponectin

Adiponectin is a negative regulator of T cells activity. Adiponectin can inhibit production of T cells through decreasing the expression of MHCII, CD80, CD86 (88) and proinflammatory cytokines, including IL-6 and TNF-α (89). Data have shown that adiponectin can inhibit Th17 cell differentiation and limit inflammation through upregulation of sirtuin 1 (SIRT1) and peroxisome proliferator-activated receptor γ (PPARγ) and suppression of RORγt (90).

#### 3.2.3 Resistin

Resistin is mainly derived from the adipose tissue. Resistin exerts proinflammatory role mostly through infiltrating immune cells in adipose tissue (91). The positive role of resistin in CD4^+^ T cells was detected in resistin-induced cell migration (92). Furthermore, proinflammatory Th17 and Th1 can be activated by resistin (91).

#### 3.2.4 IL-6

It has been reported that AT provides about 30% of circulating IL-6 (93). The level of IL-6 produced by visceral AT is higher than that of subcutaneous AT (93). It is all accepted that Th17 cells express RORγ and RORα, which are induced by TGFβ and IL-6 in a STAT3-dependent manner (94).

Obesity is a state of pathological accumulation of adipose tissue. The underlying cause of obesity and overweight is an energy imbalance between the calorie intake and the calorie consumption (95). Globally, there has been an increased intake of high-fat and high-sugar foods and an decrease in physical activity (95). Obesity in many senses is regarded as an inflammatory predisposition (96). Under white adipocyte stress, local infiltration of immune cells and enhanced production of pro-inflammatory cytokines together reduce metabolic flexibility and lead to insulin resistance in obesity (97). It actually is demonstrated in research that Th17 cells polarization is essential to maintain AT inflammation and link obesity to the development of metabolic diseases through AT inflammation (98, 99). An increase in the number of Th17 cells derived from obesity was observed in adipose tissue (83, 100, 101) or peripheral blood mononuclear cells (PBMCs) (102), which supported insulin resistance, blood glucose control and type 2 diabetes (T2D). Evidence also showed that combination of fatty acid metabolites and reduced β oxidation could stimulate Th17 inflammation in patients with T2D, which may predict the metabolic status of people with obesity (103). Additionally, increased expansion of Th17 cells significantly has been displayed to delay diabetic wound healing (104). Dendritic cells have functionality and could modulate AT inflammation *via* regulating the transformation of Th17 cell responses in obesity-associated insulin resistance (105). Obesity-induced loss of Rab4b in adipose T cells may be involved in maladaptive white adipose tissue reshaping and insulin resistance by increasing adipose Th17 (106). Furthermore, Th17 cells are characterized by production of IL-17. Study has demonstrated that IL-17 receptor knockout markedly limits the body and visceral fat pad weights of mice on HFD and ameliorates AT inflammation and insulin sensitivity (107).

When the positive energy balance is sustained and obesity progresses, a chronic, low-grade inflammatory status is established in WAT and leads to insulin resistance (97). Compared to WAT, brown adipose tissue is less susceptible to developing local inflammation in response to obesity (97). BAT-mediated thermogenesis can protect against obesity *via* promoting energy expenditure (108). However, pro-inflammatory cytokines may alter the specific thermogenic activity of BAT. Inflammation that infiltrates into BAT may both cause insulin resistance and reduce thermogenesis through weakening its energy consumption and glucose uptake capacity (109, 110).

Although mainly composed of brown adipocytes and pre-adipocytes, BAT also consists of a variety of immune cells (110). Kälin et al. (111) showed that Treg cells are critical for BAT thermogenic capacity and lipolytic function. It was believed that GRIM19 inhibited the progression of obesity by regulating BAT differentiation and the Th17/Treg balance (112). However, the relationship of BAT and Th17 cell are not mentioned. In addition, normal brown adipose tissue has the ability to regulate immunity. Moon J et al. (113) demonstrated that brown adipose tissue ameliorated autoimmune arthritis through inhibition of Th17 cells.

IFN-γ are believed to promote beige fat development. given that Th1 cells are major producers of IFN-γ, it is possible that Th1 cells may also be involved in the regulation of energy expenditure (114).

### 3.3 Th17 and Liver

The liver acts as an endocrine organ through secreting factors, which is known as hepatokines. The identification of hepatokines has greatly broadened the field of metabolic physiology. Hepatokines are liver-derived proteins who are a modulator of liver and systemic metabolism by autocrine, paracrine and endocrine signaling to affect lipid metabolism, cell function, peripheral insulin action and glucose control (19). Aside from classic hepatokines, such as adropin, angiopoietin-related proteins (ANGPTLs), fetuins and FGF21, novel hepatokines, including sex hormone-binding globulin(SHBG), apolipoprotein J (ApoJ), mesencephalic astrocyte-derived neurotrophic factor (Manf), fibronectin type III domain containing 4 (FNDC4), pregnancy zone protein (PZP) and sparc-related modular calcium-binding protein 1(SMOC1) play a key role in maintaining nutrient homeostasis by directly affecting glucose, lipid metabolism and insulin action.

Careful deduction of the Mendelian randomization results suggested that SHBG, as a hepatokine, plays a direct, causal role in the pathogenesis of female type 2 diabetes (115). Liver is the main source of circulating ApoJ. As a novel hepatokine, ApoJ aims at muscle glucose metabolism and insulin sensitivity through a low-density lipoprotein receptor-related protein-2 (LRP2)-dependent mechanism and the insulin receptor signaling cascade (116). Manf is a feeding-induced hepatokine. Manf specific overexpression in liver improved high-fat diet-induced obesity, promoted browning of subcutaneous white AT *via* the p38 MAPK pathway and also have relationship with the improvement of insulin sensitivity and hepatic steatosis (117). Liver primarily controls the circulating levels of soluble FNDC4 (sFNDC4). Data provided evidence that sFNDC4 acted as a hepatokine (118). The reduction of liver FNDC4 mRNA corresponded with sFNDC4-circulating levels, which subsequently resulted in prediabetes in mice (118). A novel hepatokine, PZP, was identified to promote diet-induced thermogenesis through activating brown adipose tissue (119).

Among those molecules, FGF21 and SMOC1, are involved in the regulation of Th17 cells.

#### 3.3.1 FGF21

FGF21 is a stress-inducible hormone that plays a crucial role in the modulation of energy balance, glucose and lipid metabolism. The liver is the main organ that controls the secretion and actions of FGF21 (120). Studies revealed that the liver regulated carbohydrate intake through production of the hepatokine FGF21 (7). It was observed that there were significant improvements of dyslipidaemia, hepatic fat fractions and serum markers of liver fibrosis in patients with non-alcoholic steatohepatitis (NASH) with administration of FGF21 (121). Hepatic inflammation was greatly reduced in NASH mice with the treatment of FGF21, which was related to suppression of IL-17A expression in Th17 cells (122). In addition, FGF21 depended on adiponectin or STAT3/RORγt pathway (123) to exert its inhibition of Th17 cell differentiation and IL-17A expression (122).

#### 3.3.2 SMOC1

SMOC1 was identified as a glucose-responsive hepatokine and a regulator of glucose metabolism, which was dysregulated in the setting of NAFLD in mice (124). However, Ghodsian et al. (125) proposed that the hepatokine SMOC1 may not regulated by NAFLD and may not modulate glucose-insulin homeostasis in humans. The different results suggest that SMOC1 in the regulation of metabolism in rodents may not always translate to human biology. Proof showed that the expression of SMOC1 was negatively correlated with levels of CD4^+^ T cells (126). Additionally, SMOC1 co-expressed genes were suppressed in processes such as adaptive immune response, T cell activation and pathways including Th17 cell differentiation (126).

NAFLD is regarded as an ectopic accumulation of fat in the liver. It is confirmed by imaging or histology that there is no known cause of secondary hepatic fat accumulation such as drinking, steatogenic drugs or genetic diseases (127). NAFLD consists of a great variety of histological spectrum ranging from benign simple steatosis to NASH. Persistent hepatic inflammation plays an essential role in this process (128). It is generally believed that changes of T cell responses and related cytokines may cause the progress of NASH. Notably, a higher frequency of Th17 cells was found in liver, which was the sign of progression from nonalcoholic fatty liver (NAFL) to NASH (129). The steatotic liver microenvironment promotes the production and metabolism of Th17 cells. Th17 cells are capable of transforming into an inflammatory hepatic CXCR3+Th17 (ihTh17) cell, which is adequate to aggravate NAFLD pathogenesis (130). The increased expression of hepatic Th17 cells and IL-17 were detected in NASH mice and patients, separately (131). Obesity promotes peripheral Th17 cell expansion and infiltration into liver (130). DNA damage in hepatocytes was found to trigger inflammation through Th17 cells and IL-17A, which induced the release of fatty acid who stored in liver as triglycerides, causing NASH (132). RNA clustering analysis of liver demonstrated that high-fat high-fructose diet-induced elevated Th17 cells in liver, especially through up-regulation of *Rorc* (coding for the canonical transcription factor RORγt) in murine nonalcoholic steatohepatitis. Moreover, pathway analysis showed enrichment of the Th17 activation pathway in liver, thus causing inflammation and NASH (72). Converted double negative T cells could limit inflammation in the liver and NASH development by lowering the ratio and survival of CD4^+^ T cell, and Th17 cell differentiation in the liver (133).

### 3.4 Th17 and Bone

The skeleton, as an endocrine organ, modulates various energy metabolism. The three main cell types in the bone, osteoblasts, osteocytes, and osteoclasts, regulate metabolism by production of molecules. Recently, multiple factors secreted by bone, known as osteokines, have been considered as regulators systemically. OCN is particularly secreted by osteoblasts, and is the most plentiful non-collagenous protein in bone (134). As a hormone, OCN was identified to inhibit bone formation and function in the regulation of glycometabolism in the pancreas, testosterone synthesis in the testis and muscle mass (134). Indeed, FGF23 play a crucial role in the modulation of other organs through being released into the circulation by osteoblasts/osteocytes, and it also create sophisticated endocrine feedback loops that regulate mineral and energy metabolism (135). Recent study demonstrated that osteoblast-derived LCN2 sustains glucose homeostasis through the regulation of glucose tolerance, insulin sensitivity, and insulin secretion (136). Sclerostin is a secreted glycoprotein primarily expressed by mature osteocytes and is widely regarded as a negative regulator of osteogenesis (137). Evidence showed that sclerostin can exert endocrine effects on stimulating adipogenesis (137). However, association between theses osteokines and Th17 cells remains to be seen.

Osteoporosis is a metabolic bone disease, which can increase the risk of fragility fractures. Osteoporosis is characterized by low bone mass and deterioration of bone microarchitecture (138). Aging and estrogen deficiency may be two of the most key risk factors in the development of osteoporosis and both of them have impact on immune function (139). Recently, the immune system and immune factors play vital roles in the occurrence and development of osteoporosis, especially in the differentiation of osteoclast (140). Large quantities of studies have demonstrated that differentiation and activation of osteoclasts that are out of control lead to bone erosion (141). Bone destruction is directly or indirectly modulated by CD4^+^ T cells that infiltrate into the lesion (142). There is a possibility that the Th lineage that is in charge of pathogenic mechanism of oestrogen-deficient osteoporosis contribute to the major sources of inflammatory cytokines to promote bone loss (143). However, as a candidate Th lineage, the Th1/Th2 pattern fails to meet those requirements (143). Studies indicated that Th17 cells play essential roles in the regulation of the process of bone reconstruction and are responsible for promoting osteoclastogenesis (140, 144). Evidence showed that elevated Th17 cell frequency and IL-17 level were related to low bone mineral density (145). Interaction between Myeloid-derived suppressor cells (MDSC) and Th17 cells potentiated concurrently abnormal inflammation and osteoclastogenesis (141). Increased proportion of anti-osteoclastogenic T lymphocytes (Treg cells) and decreased osteoclastogenic T lymphocytes (Th17 cells) were found to enhance bone health in ovariectomy mice (146). In terms of mechanism, receptor activator of nuclear factor kappa B ligand (RANKL) is a vital factor that connects the bone and immune systems (147). The stimulation of RANKL activated a signal pathway downstream of RANK, which can evaluate the degree of bone resorption *via* inducing osteoclast maturation (147). Activated T cells are primary sources of RANKL (148). Th17 cells are one of the main causes of bone loss through expressing high RANKL level (149). Besides, Th17 cells enhanced the expression of RANKL on osteoblasts and fibroblasts by the production of inflammatory cytokines, such as IL-6, IL-17 and TNF-α, and subsequently promoted osteoclast-mediated bone resorption (150). Additionally, RANKL/RANK/osteoprotegerin (OPG) system has been proposed to be of great importance to periodontitis bone metabolism, and its relationship with the Th17/Treg cell imbalance made periodontal bone metabolism and the immune system closely connected (151).

## 4 Th17 in gut-organs axis

Recently, it has been well documented that the gut may influence other organs through the production of microbiota capable of regulating immune cells. Gut microbiota may also trigger systemic immune responses through immune cell (36). Gut interacts with peripheral organs such as the adipose tissue, liver and bone to control diverse processes, including lipid metabolism, insulin resistance, bone loss, and inflammation conditions. Among immune cells, Th17 cells in draining MLNs of the gut, get into systemic circulation to promote immune responses of other organs in resistance to the same organism or other antigens in cross-reaction to similar epitopes (152). Migratory Th17 cells have remarkable plasticity in function on the basis of the existing local state (153).

### 4.1 Th17 in gut-adipose

Gut microbiota influence host metabolism *via* crosstalk with the adipose tissue, which contribute to an alteration in metabolism related to obesity. Gut could regulate several events of adipose tissue function, including lipid metabolism, endocrine function (154) and inflammation (155).

Growing evidences revealed immunological crosstalk between the gut and AT. It is found in obesity that the immunomodulatory characteristics of the gut microbiota make it attractive in the context of metainflammation (156). Obesity is related to crucial alterations in the adaptive immune compartment in the gut, which leads to abnormal inflammatory skewing (42, 157). During obesity, bowel inflammation may directly contribute to visceral AT inflammation and insulin resistance (155). Pro-inflammatory changes in immune cells, including Th17, Th1 and IL-17-producing γδ T cells, contribute to increased gut permeability (155). Lack of gut integrity allows bacterial products to flow into metabolic tissue, such as the visceral AT, where it further stimulates local immune cells and contributes to the chronic inflammation of adipose tissue that is associated with the development of insulin resistance, and other conditions related to the metabolic syndrome (158).

There are homeostatic tissue-resident and inflammatory Th17 cells with different functions in the intestine (30). In contrast to homeostatic Th17 cells, infection-induced Th17 cells in intestine displayed widespread plasticity towards pro-inflammatory cytokines, and distributed widely from intestine to the periphery (30). Diet regulates gut microbiota and Th17 cells, and plays a crucial role in the regulation of adipose tissue. A significant drop was detected in Th17 cells that accumulate in visceral adipose tissue on ketogenic diet, which may be associated with gut microbial-induced alterations in the intestinal immune environment (47). A HFD-derived ileum microbiota was found to trigger a drop in Th17 cells of the lamina propria, which contributed to metabolic inflammation by the means of transferring from the gut across the damaged intestinal barrier to diverse metabolic organs, such as the liver and adipose tissue (159, 160). Secretion of IL‐17 is mediated largely by IL‐17‐producing Th17 cells of intestinal lamina propria. IL‐17 receptor (IL‐17R) deficiency on the HFD contributed to inflammation and damage in the gut, which prevented the absorption and subsequent deposition of lipids in adipose tissue (161).

Besides, Th17 cells also play a protective role in gut and AT, and most of them are homeostatic gut-resident Th17 cells. Carlos et al. (162) proved that intestinal Th17 response limited intestinal microbiome disorder and lipopolysaccharide (LPS) translocation to visceral AT, which protected against metabolic syndrome. Gut-homing property of the transferred TH17 cells reversed the weight gain and decreased the fat mass (163).

### 4.2 Th17 in gut-liver

As 70% of its blood supply comes from the portal vein, the liver is physiologically exposed to intestinal microorganisms and metabolites (164). Th17 cells in intestinal dysbiosis are associated with liver diseases, including alcohol-associated liver disease(ALD), primary sclerosing cholangitis (PSC) and NAFLD. One hypothesis is that intestinal inflammation and barrier dysfunction are responsible for liver disease as the bacteria and inflammatory cells flow into the liver (165). Alcohol intake induced a pro-inflammatory shift in Th17 cells in the gut, which resulted in ALD *via* Sphingosine-1-Phosphate (S1P)/S1P receptor 1 (S1PR1) signaling, moreover, the decreased level of intestinal Th17 cells limited liver damage (166). PSC coexists frequently with IBD, which identifies the gut-liver axis as the core of pathogenesis (167). It also exemplifies the ‘leaky gut’ hypothesis. Gut microbiota contributes to the impairment of the first intestinal barrier, and a second trigger, such as colitis or hepatobiliary injury, further promotes the TH17-mediated disease development in PSC (168).

In parallel with the ‘leaky gut’ hypothesis, a ‘gut lymphocyte homing’ hypothesis has displayed the pathogenesis of gut–liver axis (169). Mucosal T cells produced by intestine were abnormally activated by the commensal microbiome, which further translocated to the liver and cross-reacted with antigens in the liver (169, 170). In accordance with this hypothesis, plenty of studies have been performed. Research about PSC patients with concurrent inflammatory bowel disease (PSC-IBD) clearly showed that T cells infiltration in the gut and liver of PSC-IBD patients were clonally correlative and may be able to recognize the same antigens (171). Gut-derived memory T cells that infiltrated in liver were related to enhanced Th17 cytokines (172). Co-culture assays showed MLNs CD4^+^ T cells from HFD-fed mice skewed toward migrating to the liver and contributed to hepatic inflammation (173). Adoptive transfer of MLNs CD4^+^ T cells from NAFLD mice to HFD-fed mice led to elevated transaminase, more severe hepatic inflammation and lipid accumulation (173).

### 4.3 Th17 in gut-bone

During the past 20 years, inflammatory diseases associated with gut have been proposed to be related to a drop in bone mass, which suggested that the gut may be interlinked with the bone. A growing body of evidences support an essential role of Th17 cells in gut-bone axis, especially in bone loss associated with menopause, periodontal disease, parathyroid hormone (PTH) and rheumatoid arthritis. It is obvious that the crosstalk between the immune system and the gut microbiota have extensive impact on bone health (174). Gut microbiome is responsible for pathological process of osteoporosis. The molecular mechanisms mainly include: 1) Intestinal barrier and nutrient absorption (involving short-chain-fatty acids), 2) Immunoregulation (Th17 and Treg cells balance), 3) Modulation of intestinal-brain axis (involving 5-HT) (175).

Estrogen deficiency is the etiology of postmenopausal osteoporosis (PMO). It has been widely accepted that estrogen plays an key role in the regulation of the immune system and that immune cells and associated cytokines have major influences on bone cells (176). More recent work has implicated that T cells in the gut are a proximal target of sex steroid deficiency relevant to bone loss (177). The possible mechanism is that ovariectomy increased intestinal Th17 cells and TNF^+^ T cells, enhanced their S1PR1-mediated excretion from the intestine, and further promoted their influx into the bone marrow (BM) through CXCR3- and CCL20-mediated mechanisms (177). These data emphasize the role that played by the gut microbiota in triggering intestinal Th17 cells migration that are critical for bone loss.

Bone density in vertebrae and long bone is reduced by oestrogen deficiency, which also exacerbates alveolar bone loss related to inflammatory (178). Recent evidence has suggested that gut microbiome disorder associated with oestrogen deficiency enhanced gut permeability with increased serum LPS (167). Subsequent inflammatory responses induced an imbalance of Th17/Treg cells in the bone marrow and aggravated alveolar bone loss (178).

Bone loss is a common complication of hyperparathyroidism. PTH is one of the osteoregulatory hormones that depends on gut microbiome to exert its bone catabolic and bone anabolic effects (179). It has been proved that PTH contributes to Th17 cell proliferation in the small intestine and results in bone loss (180). A specific bacterial strain, SFB, that causes Th17 cell expansion in the gut, negatively influences skeletal maturation (181). PTH only led to bone loss in mice whose gut microbiota was enriched by the Th17 cell-induced SFB (180).

Recent data indicate that osteoporosis and joint disease associated with inflammation share a joint immune component (175). Th17 cells differentiation have been shown to play a key role in rheumatoid arthritis and IBD induced bone loss (182).

## 5 Conclusion

In summary, Th17 cells are vital regulators in various endocrine organs, and contribute to crosstalk between gut and other organs, including adipose tissue, liver and bone, and eventually trigger multiple metabolic disorders and inflammatory conditions. Re-thinking about metabolic diseases relevant to endocrine organs from Th17 cells point of view may produce additional understanding of the disease mechanisms. Th17 cells have its dual roles, which are not only pathogenic in many inflammatory diseases, but also protect against extracellular bacteria, fungi, and viruses. Targeting pathological Th17 cells may be a promising treatment for systematic diseases, including IBD, obesity, insulin resistance, NAFLD, PSC and osteoporosis. Although therapies on the basis of Th17 cells are still at the initial stage of research, a great deal of studies have broadened our comprehension of Th17 cells in pathophysiology.

However, current understanding of the crosstalk between Th17 cells and endocrine system is far from enough. For example, few researches focus on the effects of osteokines on Th17 cells. Besides, plenty of factors, including diet, temperature and heredity, participate in the modulation of gut on adipose tissue. The role of Th17 cells that are influenced by temperature and heredity in gut-adipose axis is not fully expounded.

## Author contributions

CZ wrote the manuscript. DW arranged literature. LH and YG reviewed the manuscript. All authors contributed to the article and approved the submitted version.
